# Brief Report: Oxytocin Enhances Paternal Sensitivity to a Child with Autism: A Double-Blind Within-Subject Experiment with Intranasally Administered Oxytocin

**DOI:** 10.1007/s10803-012-1536-6

**Published:** 2012-04-28

**Authors:** Fabiënne B. A. Naber, Irina E. Poslawsky, Marinus H. van IJzendoorn, Herman van Engeland, Marian J. Bakermans-Kranenburg

**Affiliations:** 1 Centre of Child and Family Studies, University of Leiden, Leiden, The Netherlands; 2 Rudolph Magnus Institute of Neuroscience, Department of Child and Adolescent Psychiatry, University Medical Center Utrecht, Utrecht, The Netherlands

**Keywords:** Autism, Oxytocin, Fathers, Sensitive parenting

## Abstract

Oxytocin seems associated with parenting style, and experimental work showed positive effects of intranasally administered oxytocin on parenting style of fathers. Here, the first double-blind, placebo-controlled, within-subject experiment with intranasal oxytocin administration to fathers of children with autism spectrum disorder (ASD) is presented. Fathers with their typically developing toddler (*n* = 18), and fathers of toddlers diagnosed with ASD (*n* = 14), were observed in two play sessions of 15 min each with an intervening period of 1 week. In all fathers oxytocin elevated the quality of paternal sensitive play: fathers stimulated their child in a more optimal way, and they showed less hostility which suggests the positive effects of oxytocin on paternal sensitive play irrespective of clinical status of their child.

## Introduction

The neuropeptide oxytocin creates warm feelings for offspring (Carter 1998; Feldman et al. 2007; Galbally et al. 2011; Insel 1992) and support empathic concern for conspecifics (MacDonald and MacDonald 2010) through better recognition of emotional facial expression (Bartz et al. 2010). Moreover, it would elevate the level of trust in other human beings, in particular when they belong to the in-group (De Dreu et al. 2010; Kosfeld et al. 2005). Oxytocin administration was also shown to enhance the ability of adults with autism to understand emotions in speech (Hollander et al. 2007), and to help children with autism to better recognize people’s intentions by reading their eyes (Guastella et al. 2010).

In a recent meta-analysis on experiments with intranasally administered oxytocin in non-clinical samples it was concluded that a sniff of oxytocin indeed changes emotion perception and behavior in trusting relationships (Van IJzendoorn and Bakermans-Kranenburg 2012). In one of the experiments it was shown that fathers of typically developing toddlers showed more sensitive structuring of their play after they were administered oxytocin (Naber et al. 2010). Here we test through a double-blind within-subject experiment whether sensitive responsiveness of fathers for their child with autism can be enhanced by intranasally administered oxytocin similar to fathers of typically developing children.

Oxytocin is produced in the hypothalamus, in particular in the paraventricular nucleus of the hypothalamus, and sent down into the pituitary to reach the bloodstream as a hormone, or up into the limbic system and cortex as a neurotransmitter. Oxytocin promotes lactation (Panksepp 1998) and is also involved in enhancing the sensitivity for social and emotional signals, and lowering feelings of anxiety and stress (Carter 1998). In a study with functional magnetic resonance imaging (fMRI) it was demonstrated that oxytocin administration reduced right amygdala activation and enhanced insula and inferior frontal gyrus activation when subjects were exposed to infant crying compared to scrambled control sounds. Reduced amygdala activation may point to decreased aversive feelings to cry signals whereas increased activation of the insula and inferior frontal gyrus may reflect more empathic processing of emotional stimuli (Riem et al. 2011).

Oxytocin is not only involved in contractions during parturition and in milk production, but also in the development of parent-infant attachment, in mothers as well as in fathers. In a pioneering set of studies Feldman et al. (2007, 2010) examined oxytocin across pregnancy and the early postpartum period in relation to subsequent maternal and paternal interactive behavior to the infant. Elevated oxytocin levels were found to be associated with more sensitive parental interactions with the infant. Examining oxytocin in mothers and fathers engaged in a 15-min play-and-contact interaction session with their 4–6 month old infants Feldman et al. (2010) found similar levels of oxytocin in mothers and fathers which were associated with parent-specific modes of interaction. Whereas higher maternal oxytocin levels were associated with more affectionate touch, higher paternal oxytocin levels were uniquely associated with more stimulatory play, but not affectionate touch. Furthermore, highly affectionate mothers showed oxytocin increases following playful interactions with their infants while similar increases were observed in highly stimulating fathers.

In an experimental study with fathers of typically developing toddlers we showed that intranasal oxytocin administration leads to more responsive interactions of fathers with their child during play (Naber et al. 2010). In the oxytocin condition fathers stimulated their child’s exploration and autonomy in a better way than in the placebo condition. The fathers also tended to show less hostility to the child in the oxytocin condition showing less impatience and discontent. This experimental finding nicely converges with the Feldman et al. (2010) study that found elevated oxytocin levels in fathers who were asked to engage in a ‘‘play-and touch’’ interaction session with their infant. Both studies combined may indicate that fathers who are more responsive when interacting with their infants during play may produce more oxytocin, which in turn may increase the pleasure of the father in the interaction with the child and contribute to the father’s ability to provide responsive care of a stimulating nature.

Parenting a child requires many social skills of the parent. Parenting a child with autism seems even more complicated. For example, when a typical developing child is crying, one usually soothes the child by holding it. However, in the case of autism, holding can be experienced by the child as even more stressful. The signals of children with autism are not always easy to understand, or they trigger parental responses that are adequate for typically developing children, but not for children with autism. The difficulties in social interaction of a child with autism are often reported by parents from the first months of the child’s life, long before the child receives the diagnosis of autism (Lord 1995). It seems that the parents of a child with autism need to interact in different ways compared to parents of typically developing children. They need to be sensitive to the autistic characteristics of their child, in order to avoid intrusiveness or overstimulation.

Because fathers of children with autism are challenged even more than fathers of typically developing children in finding sensitive ways of playful interactions we examine here the effects of intranasally administered oxytocin on paternal sensitivity and structuring during play. We expect to find an increase in paternal sensitivity during play with a child with autism to a similar extent as in case of fathers of a typically developing child.

## Method

### Procedure

Eighteen fathers of typical developing children (mean age 37.4 years, SD = 4.23, range 31–45) and fourteen fathers of a child with ASD (mean age 39.4 years, SD = 6.46, range 33–45) participated in the double-blind, placebo-controlled, within-subject design. Child psychiatrists diagnosed the children as defined by the Diagnostic and Statistical Manual of Mental Disorders (DSM-IV) and the Autism Diagnostic Observation Schedule (ADOS; Lord et al. 2002). Seventeen of the 18 fathers of the typically developing children were included in a previous oxytocin experiment (see Naber et al. 2010). All participants received once intranasal oxytocin and once a placebo to investigate the effects of oxytocin on the interaction with their child. Neither the experimenter nor the participant knew during which visit the participant received oxytocin; roughly half of them (*n* = 15) received oxytocin during the first visit. The study protocol was approved by the ethics committee of the University Medical Center Utrecht. All fathers gave written informed consent before their participation. Written informed consent for the children was given by both parents. The participants were all healthy volunteers with at least one child in the age between 1.5 and 6 years of age (mean age 44.0 months, SD = 14.51). Children with ASD were older (mean age 56.14 months, SD = 7.20) compared to the typically developing children (mean age 33.41 months, SD = 10.72), *t* = −6.77, *p* < .01. A single dose of 24 IU oxytocin nasal spray (Syntocinon spray, Novartis, Basal, Switzerland) or placebo, nasal spray without oxytocin, was administered intranasally 45 min before the start of the play session. In a recent study we showed that oxytocin levels remain elevated even more than 2 h after intranasal administration (Huffmeijer et al. 2012). In the 45 min before the play session the fathers performed (computer) tasks without their child. Participants underwent both the oxytocin and the placebo conditions with an interval of one week in a balanced within-subject design.

The play sessions lasted 15 min and were slightly different on both occasions. During the first visit they played a game in which father and child in turn placed tiny dolls on a rocking tower until the tower fell over, they played with a doctor-kit, and they made a large floor-puzzle. During the second visit, father and child were invited to build a tower of magnets, after which they played with a doll and a tea-set, and then painted a coloring picture. Fathers were instructed to play with their child as they usually did. Both sessions took place at home and were videotaped for later analysis.

### Measures

#### Paternal Responsiveness

The Emotional Availability Scales (EAS; Biringen et al. 1998) were used to assess paternal sensitivity. The scales consist of four subscales for parenting behavior; sensitivity, structuring, non-intrusiveness, and non-hostility. Sensitivity of the parent was coded on a 9-point rating scale. High sensitivity refers to a parent’s responsiveness to the child’s emotional signals and communication in a flexible way. A parent was coded insensitive when interaction between parent and child was rare or inflexible. Parental structuring was coded on a 5-point rating scale. It refers to the parental ability to support learning and exploration with respect for the child’s autonomy. When a parent does not offer structure or does not involve the child in play, low scores on structuring are assigned. High scores for structuring are assigned when parents not only respond to the child’s verbal cues, but also to non-verbal cues. Non-intrusiveness was coded on a 5-point rating scale with high scores for parents who are available for the child without being interfering, overprotective, or overwhelming. An intrusive parent controls the interaction and shows lack of respect for the child’s autonomy. Non-hostility was also coded on a 5-point rating scale. Parents received high scores when no negative emotions (e.g. impatience, discontent, rolling the eyes, etc.) were shown.

The EAS also includes rating scales for child responsiveness and involvement. Responsiveness is rated as the intensity and the quality of the child’s reactions to the parent’s bids, while involvement reflects the child’s attempts to engage the parent in the interaction. Child responsiveness and child involvement were coded on 7-point rating scales.

The play sessions were coded by three trained observers, who were unaware of the condition of the fathers (oxytocin or placebo). Two of them coded the fathers of typically developing children (mean intra-class correlation for the parenting scales *r* = .89, *n* = 11); and two of them (one overlapping) coded the fathers of children with autism (mean intra-class correlation *r* = .78, *n* = 7). We used one observer’s ratings of all first visit play sessions and the other’s ratings of all second visit play sessions.

## Results

**Fig. 1:**
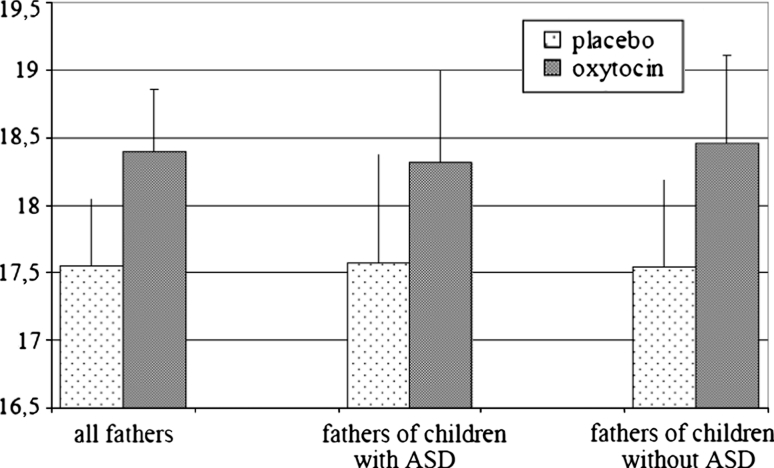
Scores (M, SE) on the overall parenting scale in the placebo and oxytocin conditions for the total group, for fathers of children with ASD, and for fathers of typically developing children

A repeated measures analysis of variance with the overall parenting scale as dependent variable, condition (oxytocin or placebo) as a within-subject factor, and child gender and group (typically developing children or children with ASD) as between-subject factors showed a significant effect for condition, *F*(1, 29) = 4.18, *p* = .050, *η* ^2^ = .13. Group and gender were no significant predictors (group, *F*(1, 29) = 0.02, *p* = .879, *η* ^2^ < .01; gender, *F*(1, 29) = 0.06, *p* = .813, *η* ^2^ < .01). The interaction effects of condition and group, *F*(1, 29) < 0.01, *p* = .99, *η* ^2^ < .01, and of condition and gender, *F*(1, 29) = 0.20, *p* = .66, *η* ^2^ = .01, were not significant, implying that the effect of oxytocin was similar in fathers of normally developing children and in fathers of children with ASD, and similar in fathers of boys and fathers of girls. Figure 1 shows the scores on the overall parenting scale in the placebo and oxytocin conditions for the total group, for fathers of children with ASD, and for fathers of typically developing children.

A multivariate repeated measures analysis of variance on the four parenting scales with condition (oxytocin or placebo) as a within-subject factor showed an overall effect of oxytocin administration on parenting behavior *F*(4, 28) = 2.74, *p* = .048, *η* ^2^ = .28. Univariate analyses showed significant effects for *Structuring*, *F*(1, 31) = 8.33, *p* = .007, *η* ^2^ = .21, and for *Hostility*, *F*(1, 31) = 6.13, *p* = .019, *η* ^2^ = .17. Effects for *Sensitivity*, *F*(1, 31) = 0.93, *p* = .34, *η* ^2^ = .03, and *Intrusiveness*, *F*(1, 31) = 1.94, *p* = .17, *η* ^2^ = .06, were not significant. In the oxytocin condition fathers of normally developing and ASD children showed more structuring sensitivity and less hostility than in the placebo condition.

In a multivariate repeated measures analysis of variance on child responsiveness and child involvement with condition (oxytocin or placebo) as a within-subject factor, and child gender and group (typically developing children or children with ASD) as between-subject factors, we found a significant effect for group, *F*(2, 27) = 4.75, *p* = .017, *η* ^2^ = .25. Children with ASD were significantly less involved than typically developing children (oxytocin: ASD *M* = 4.00, SD = 2.38, typically developing *M* = 5.78, SD = 0.97; placebo: ASD *M* = 3.77, SD = 2.22, typically developing *M* = 5.64, SD = 0.76), and children with ASD showed lower levels of responsiveness (oxytocin: ASD *M* = 4.18, SD = 2.11, typically developing *M* = 5.75, SD = 0.86; placebo: ASD *M* = 4.30, SD = 1.91, typically developing *M* = 5.69, SD = 0.66). There were no significant effects for gender (*F*(2, 27) = 0.02, *p* = .976, *η* ^2^ < .01) or condition (*F*(2, 27) = 0.62, *p* = .547, *η* ^2^ = .04), nor were interaction effects between group and condition or between group and gender significant.

## Discussion

This is the first experimental study showing that intranasal oxytocin administration enhances the quality of playful interactions of fathers with their child with an autism spectrum disorder. In the oxytocin condition, fathers of children with ASD stimulated their child in a more optimal way, and they showed less hostility. The effects of oxytocin administration were similar for both fathers of typically developing children and fathers of children with ASD. Children’s involvement and responsiveness were not affected by oxytocin administered to their parent. As expected children with ASD showed lower involvement and responsiveness compared to typically developing children. Taken together these findings suggest the positive effects of oxytocin on paternal sensitive play irrespective of clinical status of their child.

The role of fathers in parenting responsibilities has increased in the last few decades (Yeung et al. 2001). In particular, family sociologists report an increased participation in positive engagement activities for fathers (Pleck 2010). However, father-child interaction seems different from mother–child interaction. Fathers are more likely to play when they interact with their child, whereas mothers spend more time in caregiving activities (Roggman et al. 2004), and this ‘specialisation’ seems reflected in the oxytocin effects we found for paternal stimulatory play. Traditionally fathers have been described as focused on stimulating play, with less emphasis on emotional support and warmth (Grossmann et al. 2008). Mothers might relate to their infants with sensitive warmth, whereas fathers might choose sensitive stimulation as a way to promote feelings of security in their infants (Lucassen et al. 2012), and it is this playful stimulation that was enhanced by oxytocin administration.

Children with ASD render their parents’ task of deciphering their signals more difficult because they may not express their emotions in explicit ways (Van IJzendoorn et al. 2007). This, together with the passivity of the child during interaction, may also confuse parents about their child’s intellectual abilities and potentials (Bouma and Schweitzer 1990; Koegel et al. 1992), which may trigger mothers of children with autism to use more physical contact, more high intensity behaviors and fewer social verbal approaches (Doussard-Roosevelt et al. 2003). Moreover, parents of children with autism more often use control strategies than parents of typically developing children (Kasari et al. 1988). When this happens in a sensitive way, attuned to the needs of the child, this may improve the developmental outcomes of the child. Our results, showing an improvement of fathers’ sensitive structuring of play interactions after oxytocin administration, may thus have important implications for the developmental outcomes of the child.

Several intervention studies have been conducted with children with autism, using a wide range of therapies (Rogers and Vismara 2008; Ospina et al. 2008). The potential benefits of parent training are increased parenting skills, renewed confidence and reduced stress for parents as well as children (McConachie and Diggle 2007). Evidence for the potentially important role of oxytocin in human parenting may be derived from experimental studies administering oxytocin to individuals with autism, which enhanced their social cognitions and empathic feelings (Bartz and Hollander 2006; Bartz et al. 2011), and in studies relating autism or parental sensitivity to functional variations in the oxytocin receptor gene (Bakermans-Kranenburg and Van IJzendoorn 2008; Jacob et al. 2007; Wu et al. 2005; Ylisaukko-oja et al. 2006). In non-clinical individuals intranasal oxytocin administration increased feelings of interpersonal trust (Heinrichs et al. 2003; Kosfeld et al. 2005), and improvement of “mind-reading” capacities (Domes et al. 2007). In the study of Hurlemann et al. (2010) it was demonstrated that oxytocin can facilitate socially reinforced learning as well as emotional empathy in men. Although our sample size was rather small we present here a first proof of principle that oxytocin levels positively influence the parenting skills of fathers of children with ASD. The effect size was substantial; note that the effect of oxytocin administration on parenting was of the order of the difference in child involvement and responsiveness between children with and without ASD. Although this finding does not necessarily imply any pharmacotherapeutic consequence (Van IJzendoorn and Bakermans-Kranenburg 2012), we suggest that the use of oxytocin as a catalyst of interaction-focused parent training might be worthwhile to examine in more detail in the next future.
